# The association between hematologic traits and aneurysm-related subarachnoid hemorrhage: a two-sample mendelian randomization study

**DOI:** 10.1038/s41598-024-62761-1

**Published:** 2024-05-22

**Authors:** Kang Peng, Abraham Ayodeji Adegboro, Yanwen Li, Hongwei Liu, Biao Xiong, Xuejun Li

**Affiliations:** 1 Department of Radiology, Xiangya Hospital, Central South University, 87 xiangya road, Changsha, Hunan China; 2 Department of Neurosurgery, Xiangya Hospital, Central South University, 87 xiangya road, Changsha, Hunan China; 3 Hunan International Scientific and Technological Cooperation Base of Brain Tumor Research, Xiangya Hospital, Central South University, Changsha, China; 4 Department of Neurosurgery, People’s Hospital of Wangcheng District, Changsha, 410200 Hunan China

**Keywords:** GWAS, Hematologic traits, Intracranial aneurysm, Mendelian randomization, Subarachnoid hemorrhage, Neuroscience, Neurology

## Abstract

Several hematologic traits have been suggested to potentially contribute to the formation and rupture of intracranial aneurysms (IA). The purpose of this study is to explore the causal association between hematologic traits and the risk of IA. To explore the causal association between hematologic traits and the risk of IA, we employed two-sample Mendelian randomization (MR) analysis. Two independent summary-level GWAS data were used for preliminary and replicated MR analyses. The inverse variance weighted (IVW) method was employed as the primary method in the MR analyses. The stabilities of the results were further confirmed by a meta-analysis. In the preliminary MR analysis, hematocrit, hemoglobin concentration (*p* = 0.0047), basophil count (*p* = 0.0219) had a suggestive inverse causal relationship with the risk of aneurysm-associated subarachnoid hemorrhage (aSAH). The monocyte percentage of white cells (*p* = 0.00956) was suggestively positively causally correlated with the risk of aSAH. In the replicated MR analysis, only the monocyte percentage of white cells (*p* = 0.00297) remained consistent with the MR results in the preliminary analysis. The hematocrit, hemoglobin concentration, and basophil count no longer showed significant causal relationship (*p* > 0.05). Meta-analysis results further confirmed that only the MR result of monocyte percentage of white cells reached significance in the random effect model and fixed effect model. None of the 25 hematologic traits was causally associated with the risk of unruptured intracranial aneurysms (uIA). This study revealed a suggestive positive association between the monocyte percentage of white cells and the risk of aSAH. This finding contributes to a better understanding that monocytes/macrophages could participate in the risk of aSAH.

## Introduction

Aneurysm-associated subarachnoid hemorrhage (aSAH) is a fetal subtype of stroke that can cause death in 25–30% of patients within 3 months of onset and result in permanent neurological dysfunction in approximately 40% of patients ^1–3^. Therefore, it is clinically important to determine the risk factors for the formation and rupture of intracranial aneurysms (IA).

Several modified risk factors, including smoking, high body mass index (BMI), elevated triglycerides level (TG), hypertension, heavy alcohol consumption, sleep apnea, and low levels of low-density lipoprotein (LDL), have been suggested to be associated with an increased risk of IA and aSAH^4–7^. Additionally, elevated serum magnesium concentration has been reported to be associated with a reduced risk of IA and aSAH^8^. However, the risk factors contributing to IA formation and aSAH are not yet thoroughly understood.

Blood components play a crucial role in maintaining oxygen transport, hemostasis, immune response, and other physiological activities^9–12^, and abnormalities in blood components have been confirmed to be associated with various diseases^13^. Recently, a Mendelian randomization (MR) study demonstrated a causal association between increased plateletcrit and eosinophil percentage of white cells and ischemic stroke^14^. Observational studies have also shown associations between abnormalities in blood components and the risk of IA formation, aSAH, and patient prognosis after aSAH^15–20^. However, the determination of whether blood components abnormalities are the cause or consequence of IA formation and aSAH is challenging due to possible residual confounding and reverse causality inherent in observational studies^21^.

Mendelian Randomization (MR) is a method that utilizes exposure-associated genetic variants as instrumental variables (IVs) to study causal associations between exposure factors and outcomes^22,23^. The advantage of using genetic variants is that it mitigates potential residual confounding and reverse causality present in observational studies. The availability of genome-wide association studies (GWAS) has provided an opportunity to explore the causal relationship between hematologic traits and IA formation and aSAH^13,24^. Therefore, the objective of our study was to examine whether hematologic traits are causally associated with the risk of IA formation and its rupture using a two-sample MR analysis.

## Methods

### GWAS summary-level data of hematologic traits

We obtained summary-level data of hematologic traits from a previous GWAS study, which included 173,480 participants without any blood disorders from the European population^13^. Twenty-five hematologic traits were analyzed, including 11 red blood cell (RBC) traits (hematocrit, hemoglobin concentration, high light scatter reticulocyte (HLSR) counts, high light scatter reticulocyte percentage of red cells (HLSR/RBC), immature fraction of reticulocytes (IFR), mean corpuscular hemoglobin (MCH), MCH concentration, mean corpuscular volume (MCV), RBC counts, reticulocyte count, and reticulocyte fraction of red cells), 10 white blood cell traits (white blood cell counts, basophil counts, eosinophil counts, lymphocyte counts, monocyte counts, neutrophil counts, basophil percentage of white cells, eosinophil percentage of white cells, neutrophil percentage of white cells, and monocyte percentage of white cells), and four platelet traits (platelet count, platelet distribution width (PDW), plateletcrit and mean platelet volume (MPV)). Single-nucleotide polymorphisms (SNPs) associated with these traits below the genome-wide significance threshold (*p* < 5 × 10^–8^) were selected as candidate instrumental variables (IVs). To ensure independence among the candidate IVs, we performed linkage disequilibrium clumping (r^2^ = 0.001, kb = 10,000), and excluded dependent candidate IVs. We harmonized the candidate IVs with the outcome data to ensure consistent effects of each SNP on the exposure and outcome. Additionally, we calculated the F-statistic for each SNP using the Formula:

$$ F = R^{{2}} \left( {n - { 2}} \right)/\left( {{1 } - R^{{2}} } \right), $$

where *n* is the sample size and *R*^2^ is the proportion of exposure variance divided by genetic variance. And we removed those SNPs with an F-statistic less than 10 to ensure sufficient instrumental strength for the exposure^25^.

### GWAS summary-level data of aSAH and uIA

For the preliminary MR analysis, we obtained the summary-level data of aSAH and unruptured intracranial aneurysm (uIA) from a previous GWAS study with European population samples, comprising 5140 aSAH cases, 2070 uIA cases, 71,934 controls, and 4,471,083 SNPs^24^.

For the replicated MR analysis, we obtained another independent summary-level data of aSAH from the FinnGen cohort (https://www.finngen.fi/en), increasing the total sample size to 377,277 cases, including 5753 aSAH cases, and 20,175,454 SNPs^26^.

### Mendelian randomization and sensitivity analyses

In this MR study, we hypothesized that the genetic variants serving as IVs were strongly associated with hematologic traits, not confounded by other factors, and solely related to the risk of aSAH or uIA through hematologic traits.

All analyses were performed using the RStudio (version 4.2.1) with the “Two Sample MR”, “Mendelian Randomization Pleiotropy Residual Sum and Outlier (MR-PRESSO)” packages. To assess the causal relationship between hematologic traits and the risk of aSAH or uIA, we conducted two-sample MR analysis using the inverse variance regression weighted (IVW) method, a widely used approach in MR analysis^27^. First, we conducted the preliminary MR analysis to identify exposure traits potentially associated with aSAH or uIA. Subsequently, we validated the causal association between the filtered exposure traits and aSAH using the FinnGen cohort.

We considered suggestive statistical significance when 0.05 > *p* > 0.002, and statistical significance was considered when *p* < 0.002 (0.05/25) after Bonferroni correction.

For sensitivity analysis, we assessed heterogeneity in the MR analysis using Cochran's *Q* test. We used both fixed effects of IVW method and MR-Egger regression to examine the causal estimates and *p-value*. If heterogeneity was detected (*p* < 0.05), then MR-PRESSO was performed to detect and correct for potential outliers due to horizontal pleiotropy^28^. We also evaluated directional pleiotropy of the IVs through MR-Egger regression analysis, where the intercept of MR-Egger regression indicated the presence or absence of directional pleiotropy^29^. Furthermore, we conducted leave-one-out sensitivity analysis to assess the stability of the MR results. This analysis involved systematically excluding each SNP individually, followed by performing MR analysis on the remaining SNPs to detect potential outliers and ensure the stability of results^30^.

### Informed consent and Ethical approval

Participants were informed, and relevant ethical approval was obtained for the original studies.

## Results

### Causal effects of hematologic traits on aSAH and uIA in the preliminary MR analysis

**Figure 1:**
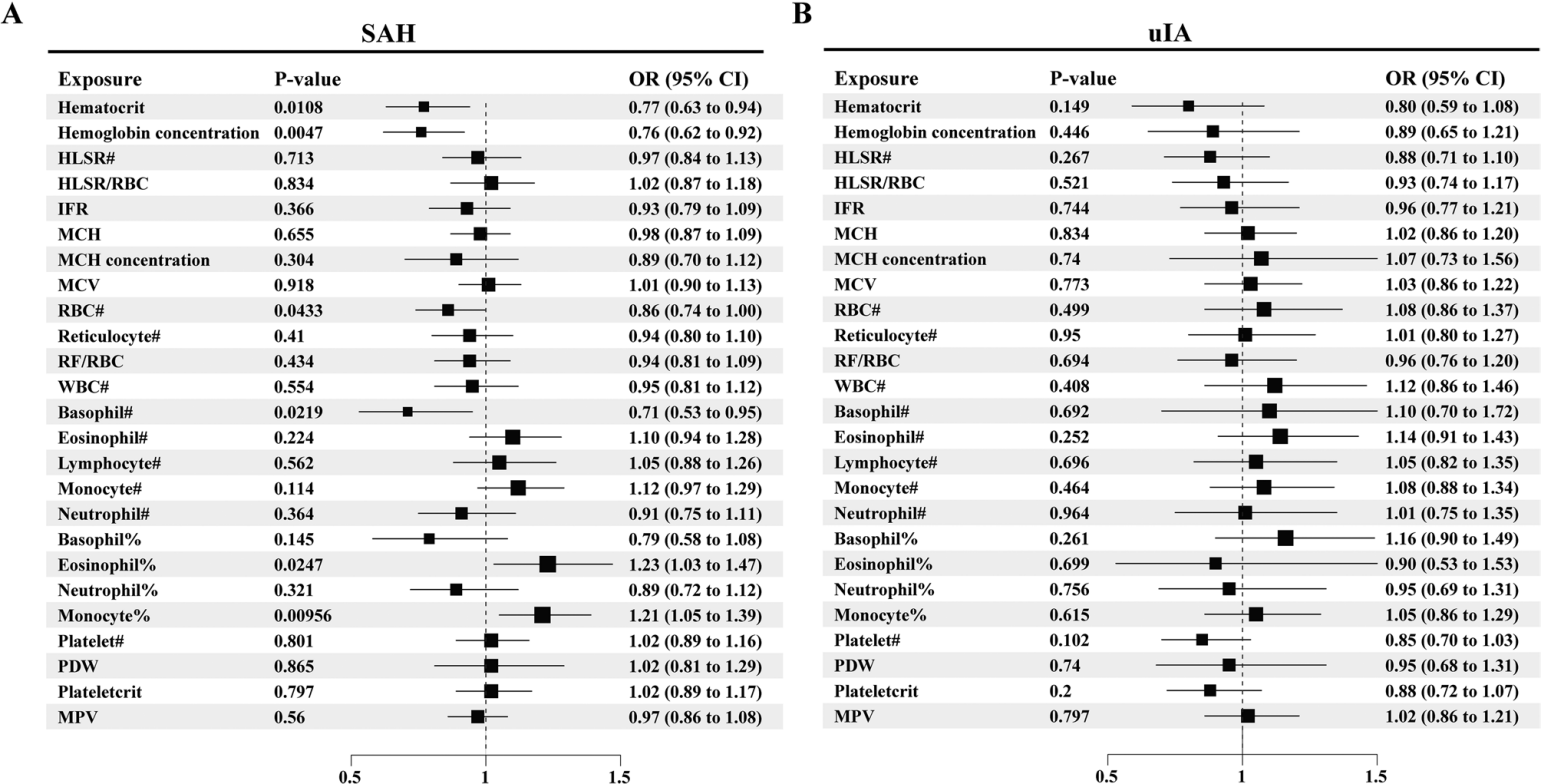
Forest plot illustrating the causal effects of 25 hematologic traits on aSAH (**A**) and uIA (**B**) in the preliminary MR analysis. The IVW method was used as the primary analysis approach. All results are presented as odds ratios (OR) along with their corresponding 95% confidence intervals (95%CI). #: counts; %: percentage of white cells.

In the preliminary MR analysis, we investigated the causal relationship between 25 hematologic traits and the occurrence of aSAH or uIA using the outcome summary statistics from the study conducted by Bakker et al.^24^. We initially discovered that hematocrit (IVW: OR = 0.77, 95%CI: 0.63–0.94, *p* = 0.0108), hemoglobin concentration (IVW: OR = 0.76, 95%CI: 0.62–0.92, *p* = 0.0047), RBC count (IVW: OR = 0.86, 95%CI: 0.74–1.00, *p* = 0.0433), basophil count (IVW: OR = 0.71, 95%CI: 0.53–0.95, *p* = 0.0219), eosinophil percentage of white cells (IVW: OR = 1.23, 95%CI: 1.03–1.47, *p* = 0.0247), and monocyte percentage of white cells (IVW: OR = 1.21, 95%CI: 1.05–1.39, *p* = 0.00956) were suggestively causally associated with the risk of aSAH (Fig. 1A). However, the remaining 19 hematologic traits were not associated with aSAH (Fig. 1A). And there was no association between the 25 hematologic traits and uIA (Fig. 1B). We also used other methods to evaluate whether there were causal relationships between hematocrit, hemoglobin concentration, RBC count, basophil count, eosinophil percentage of white cells, monocyte percentage of white cells and the risk of aSAH, but there was no significance observed (Supplementary materials: Table S1).

**Figure 2:**
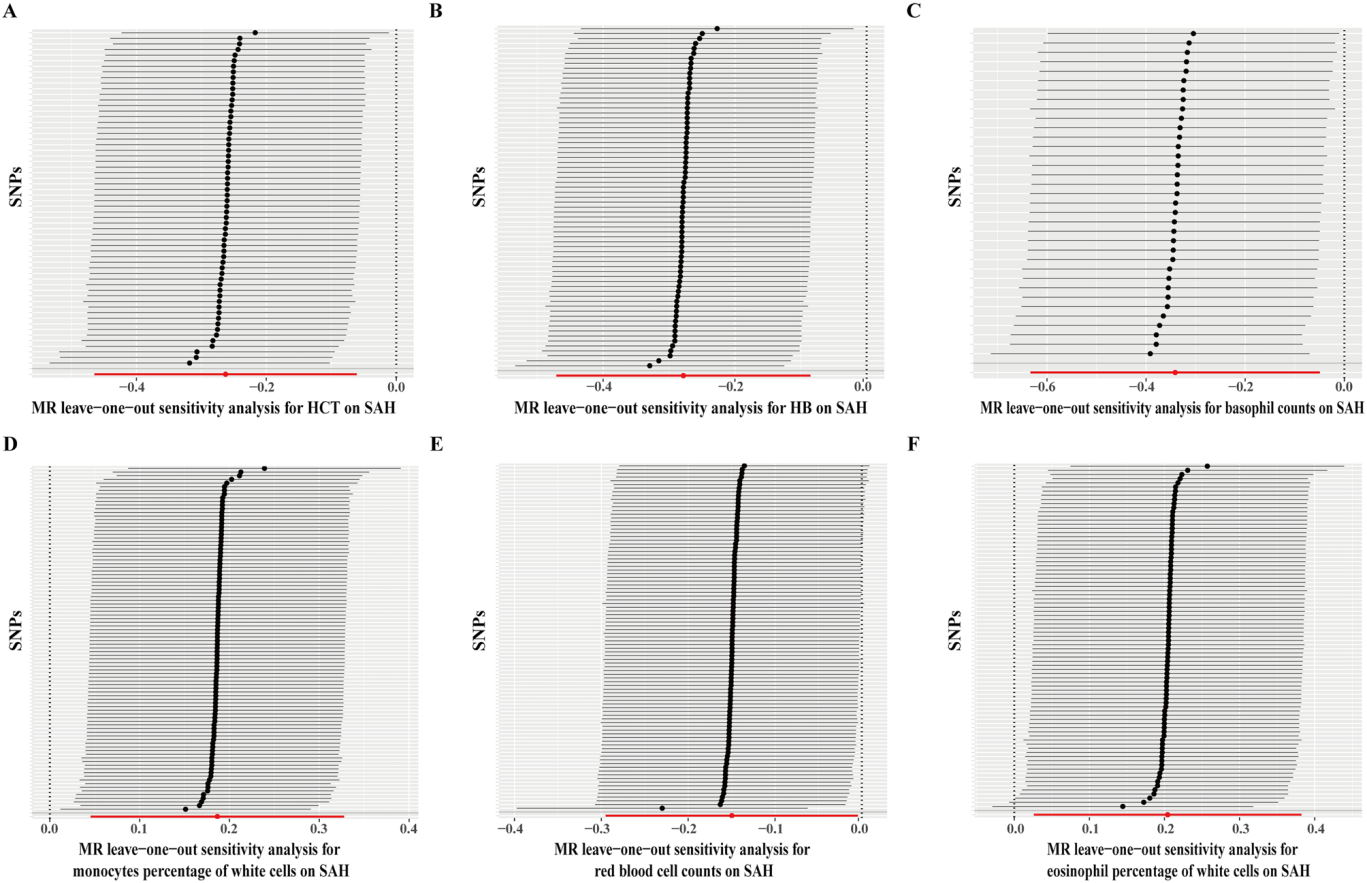
leave-one-out analysis of the MR results of hematocrit (HCT) (**A**), hemoglobin concentration (HB) (**B**), basophil counts (**C**), monocytes percentage of white cells (**D**), red blood cell counts (**E**), and eosinophil percentage of white cells (**F**) on aSAH in the preliminary MR analysis.

Next, we assessed the heterogeneity and directional pleiotropy in the preliminary MR analysis, and found no evidence of heterogeneity or directional pleiotropy (Supplementary materials: Table S2 and Table S3). Subsequently, a leave-one-out analysis was conducted to evaluate the stability of the six hematologic traits that showed potential causal associations with the risk of aSAH. As depicted in Fig. 2, none of the SNPs significantly affected the stability of the MR results for hematocrit, hemoglobin concentration, basophil count, and monocyte percentage of white cells in relation to aSAH. However, the MR results for RBC count (Fig. 2E) and eosinophil percentage of white cells (Fig. 2F) were found to be unstable. Consequently, RBC count and eosinophil percentage of white cells were excluded, and the four remaining hematologic traits (hematocrit, hemoglobin concentration, basophil count, and monocyte percentage of white cells) were selected for further replicated MR analysis and meta-analysis.

**Figure 3:**
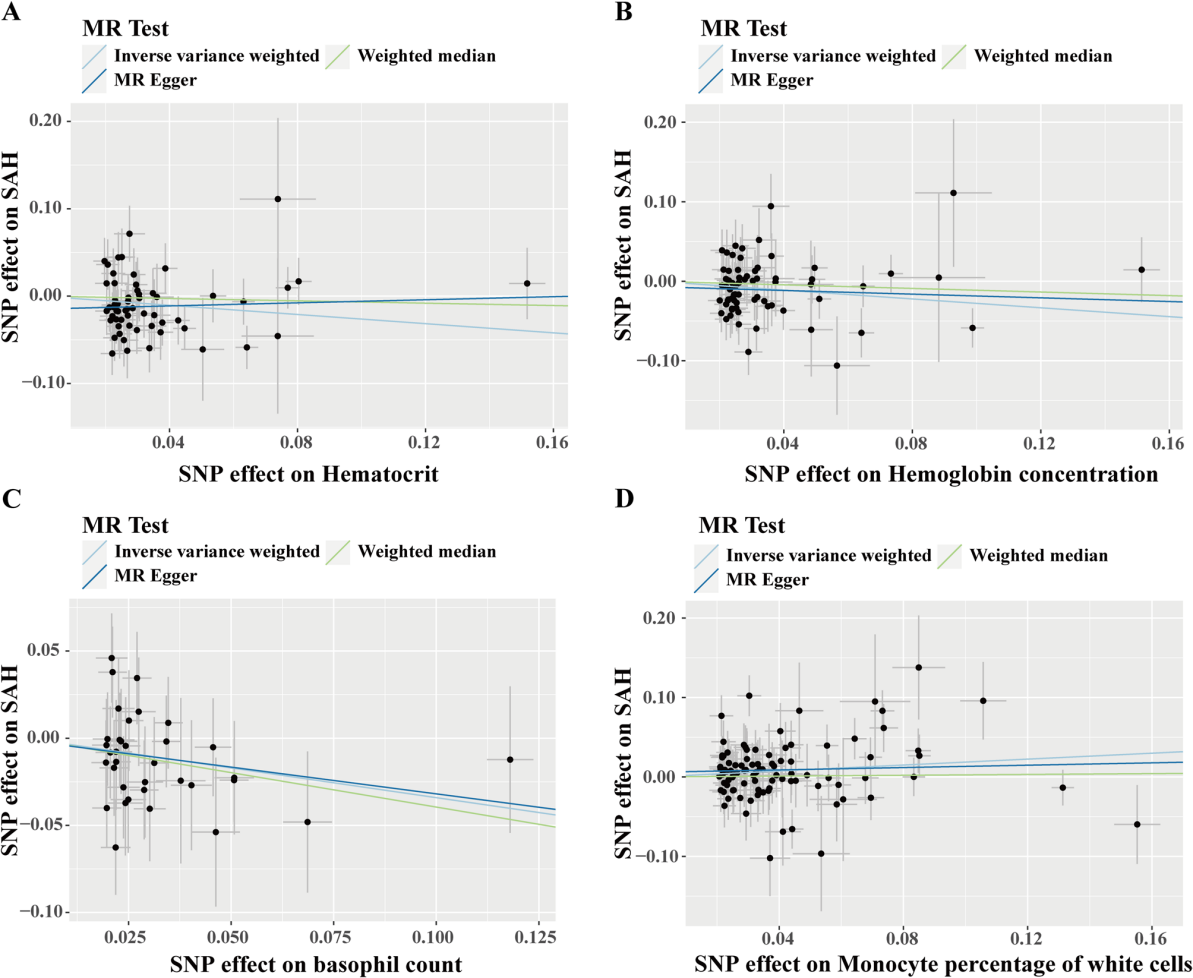
Scatter plot of the MR results of hematocrit (**A**), hemoglobin concentration (**B**), basophil count (**C**), and monocyte percentage of white cells (**D**) on the risk of aSAH in the preliminary MR analysis.

Next, we plotted the scatter plots of each SNP of hematocrit, hemoglobin concentration, basophil count, and monocyte percentage of white cells on aSAH. The scatter plots showed that hematocrit (Fig. 3A), hemoglobin concentration (Fig. 3B), and basophil count (Fig. 3C) were negatively correlated to aSAH, and monocyte percentage of white cells (Fig. 3D) was positively correlated to aSAH.

### Replicated MR analysis validates causal association between monocyte percentage of white cells and risk of aSAH

**Figure 4:**
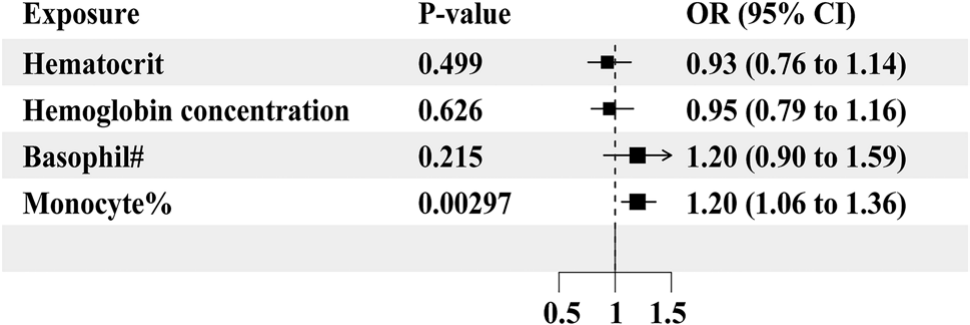
Forest plot illustrating the causal effects of hematocrit, hemoglobin concentration, basophil counts, and monocytes percentage of white cells on aSAH in the replicated MR analysis. The IVW method was used as the primary analysis approach. All results are presented as OR along with their corresponding 95%CI. #: counts; %: percentage of white cells.

**Figure 5:**
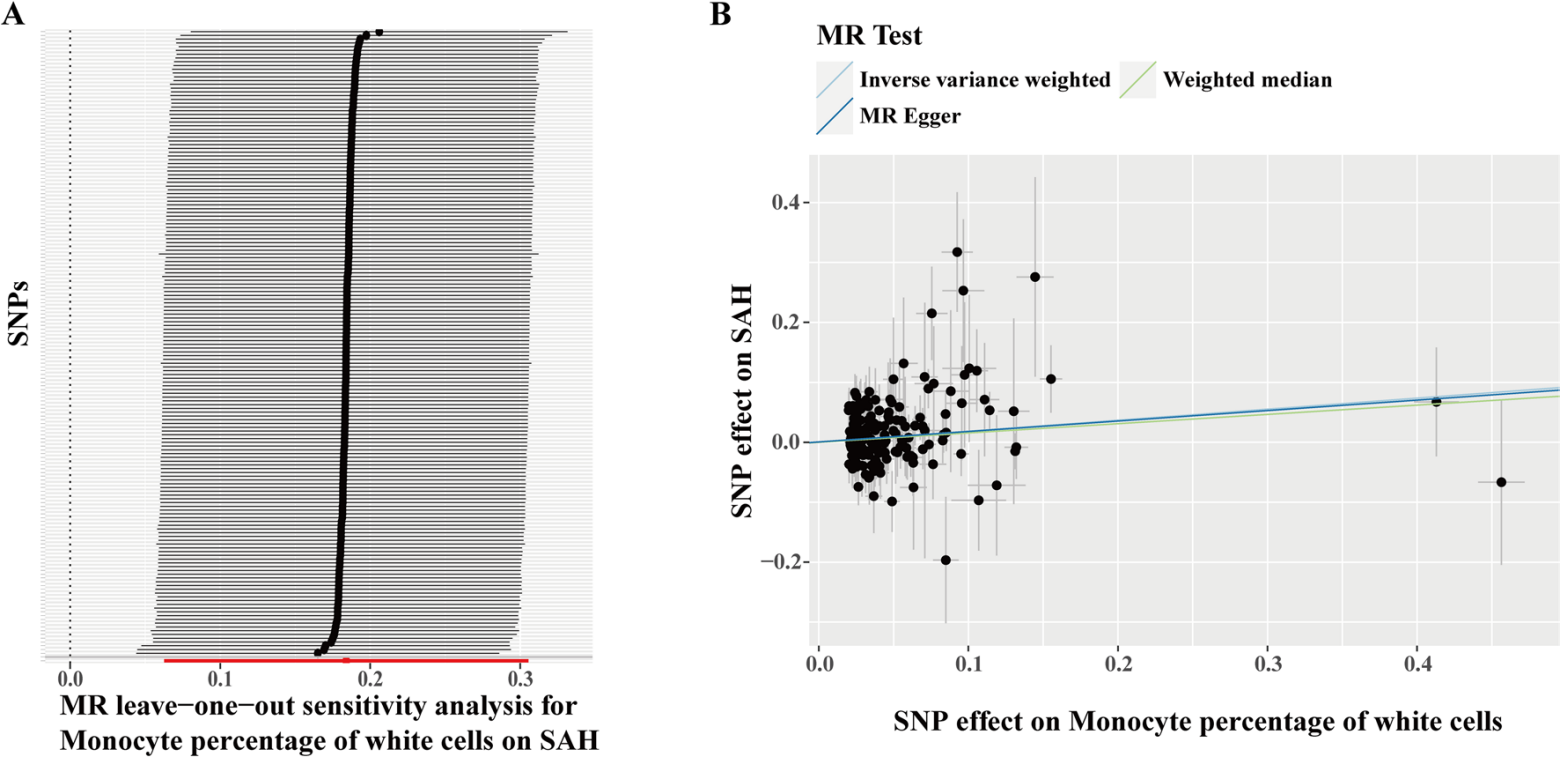
Leave-one-out analysis (**A**) and scatter plot (**B**) of the MR results of monocytes percentage of white cells on aSAH in the replicated MR analysis.

To ensure the reliability of this MR study, we collected another independent GWAS summary-level data of aSAH from the FinnGen cohort and performed MR analyses to examine the relationship between the remaining four hematologic traits and the risk of aSAH. In the replicated MR analysis, we observed that only the monocyte percentage of white cells remained significantly associated with the risk of aSAH (IVW: OR = 1.20, 95%CI: 1.06–1.36, *p* = 0.00297), hematocrit (IVW: OR = 0.93, 95%CI: 0.76–1.14, *p* = 0.499), hemoglobin concentration (IVW: OR = 0.95, 95%CI: 0.79–1.16, *p* = 0.626), and basophil count (IVW: OR = 1.20, 95%CI: 0.90–1.59, *p* = 0.215) were not causally associated with the risk of aSAH (Fig. 4). We also used other methods to evaluate whether there were causal relationships between hematocrit, hemoglobin concentration, basophil count, monocyte percentage of white cells and the risk of aSAH, there still was no significance observed (Supplementary materials: Table S4). No heterogeneity or directional pleiotropy was observed in the replicated MR analyses (Supplementary materials: Table S5). The leave-one-out analysis indicated that none of the individual IVs caused instability in the MR results for the association between monocyte percentage of white cells and aSAH (Fig. 5A). The scatter plots of each SNP of monocyte percentage of white cells also showed a positive correlation with aSAH in the replicated MR analysis (Fig. 5B).

### Meta-analysis of the preliminary and replicated MR analysis results further confirmed the causal association between monocyte percentage of white cells and risk of aSAH

**Figure 6:**
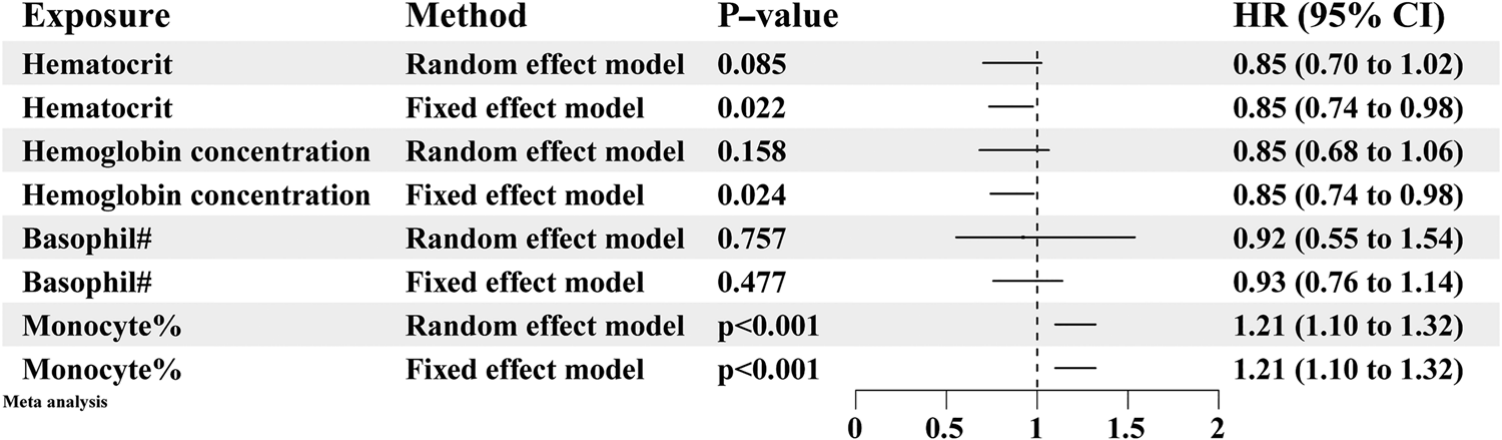
Forest plot of the meta-analysis of the preliminary and replicated MR analysis results. Both random effect method and fixed effect model were used in the meta-analysis. All results are presented as OR along with their corresponding 95%CI. #: counts; %: percentage of white cells.

We employed both random effect model and fixed effect model for the meta-analysis. As shown on Fig. 6, hematocrit was not causally associated with the risk of aSAH in the random effect model (OR = 0.85, 95%CI: 0.70–1.02, *p* = 0.085), whereas it was causally associated with the risk of aSAH in the fixed effect model (OR = 0.85, 95%CI: 0.74–0.98, *p* = 0.022); hemoglobin concentration was not causally associated with the risk of aSAH in the random effect model (OR = 0.85, 95%CI: 0.68–1.06, *p* = 0.158), but it was causally associated with the risk of aSAH in the fixed effect model (OR = 0.85, 95%CI: 0.74–0.98, *p* = 0.024); basophil count was not causally associated with the risk of aSAH in both models (random effect model: OR = 0.92, 95%CI: 0.55–1.54, *p* = 0.757; fixed effect model: OR = 0.93, 95%CI: 0.76–1.14, *p* = 0.477); monocyte percentage of white cells was causally associated with the risk of aSAH in both models (random effect model: OR = 1.21, 95%CI: 1.10–1.32, *p* < 0.001; fixed effect model: OR = 1.21, 95%CI: 1.10–1.32, *p* < 0.001).

## Discussion

In this study, we performed two-sample MR analyses to investigate the causal relationship between 25 genetically determined hematologic traits and aSAH or uIA in the European population. The preliminary MR analysis revealed suggestive causal associations between hematocrit, hemoglobin concentration, basophil count, and monocyte percentage of white cells with the risk of aSAH, while the remaining 21 hematologic traits showed no causal association. None of the 25 hematologic traits showed a causal association with uIA.

In the replicated MR analysis, we focused on the four hematologic traits that exhibited suggestive causal associations with aSAH in the preliminary analysis. However, only the monocyte percentage of white cells demonstrated a consistent causal association with the risk of aSAH. The previously observed causal associations with hematocrit, hemoglobin concentration, and basophil count did not reach significance in this independent dataset. Furthermore, only the monocyte percentage of white cells reached significance in the meta-analysis using both random effect method and fixed effect modelling.

Hematocrit and hemoglobin concentration are commonly used clinical indicators to assess anemia in patients^31,32^. A previous clinical trial demonstrated the association between anemia following aSAH and poor long-term neurological function and patient mortality^16^. Preoperative anemia has also been identified as an independent risk factor for perioperative complications and prolonged hospital stay in patients with IA undergoing surgical intervention^33^. Lower hemoglobin levels have been linked to an increased risk of acute epilepsy after aSAH^34^. Furthermore, patients with sickle cell anemia have a higher incidence of aSAH compared to the general population, and these individuals exhibit varying degrees of anemia^35^.

In our study, we initially observed an inverse causal association between hematocrit, hemoglobin concentration, and the risk of aSAH in the preliminary MR analysis. However, these causal associations did not persist in the replicated MR analysis. The inconsistent results could be attributed to variations in data sources, including differences in sequencing platforms and depths. Notably, a large Swedish cohort study reported that while anemia was a predictor of major bleeding events, (gastric/duodenal bleeding, any severe bleeding), it was not associated with intracranial hemorrhage^36^.

Based on our findings, we conclude that hematocrit and hemoglobin concentration are not causally associated with aSAH in this study. Further investigations leveraging the advancements in GWAS technology are warranted to better understand these relationships.

The inflammatory response has been implicated in the pathogenesis of IA and its rupture^37,38^. In this MR study, we aimed to explore the relationship between peripheral blood inflammatory indicators and the risk of aSAH and uIA. Our analysis revealed that only the monocyte percentage of white cells was causally associated with the risk of aSAH. Although the basophil count showed a suggestive causal association with aSAH in the preliminary MR analysis, this significance did not persist in the replicated MR analysis. Furthermore, none of the inflammatory indicators of peripheral blood were found to be causally associated with uIA.

Previous studies have demonstrated the presence of macrophages in the wall of IA, particularly in ruptured IAs in humans^12^. Macrophage infiltration has been linked to the loss of smooth muscle cells and degradation of matrix proteins in the IA wall, thereby increasing the risk of aneurysm rupture. In rodent models of IA, macrophages have also been observed in the IA wall, particularly CD68-positive macrophages^39^. Studies inhibiting monocyte chemotactic protein-1 (MCP-1), which plays a role in monocyte and eosinophil chemotaxis, have shown a marked reduction in IA occurrence and enlargement in rats^40^. Depleting macrophages using clodronate liposomes has also been shown to reduce IA formation in mice^18^. These findings collectively highlight the crucial role of monocytes/macrophages in the development and rupture of IA. Our findings in this study are consistent with previous research, supporting the notion that monocyte/macrophages play a significant role in the occurrence and rupture of IA. Although the monocyte count in peripheral blood was not causally associated with the risk of aSAH in our analysis, it further emphasizes the complex involvement of immune cells in the pathogenesis of IA.

Additionally, a recently published study demonstrated that neutrophils promote IA rupture through the formation of extracellular traps in mice^41^. Another clinical study reported higher plasma myeloperoxidase (MPO) concentration in the aneurysm compared to the femoral artery in patients with IA after endovascular coiling^42^. Moreover, the number of MPO-positive cells in IA wall was higher than in the superficial temporal artery^42^. However, in our MR analysis, both the neutrophil count and neutrophil percentage of white cells in peripheral blood were not causative factors for the formation of uIA or the risk of aSAH. These contradictory results may be due to the fact that the IVs used in this MR analysis study only represent the levels of neutrophil count or neutrophil percentage of white cells in peripheral blood and do not directly reflect the conditions in the aneurysm itself.

It is important to acknowledge several limitations in our MR analysis study. Firstly, MR analysis estimates lifelong effects rather than acute effects of exposures on outcomes. Secondly, our study focused on the European population, and further investigations are needed to explore the generalizability of these findings to other populations. Thirdly, the IVs used in this study only represented peripheral blood indicators and may not fully capture the specific characteristics of blood within the aneurysm. Differences between peripheral blood and aneurysmal blood could potentially influence the results.

Despite these limitations, our study contributes to the understanding of the role of hematologic traits and inflammatory indicators in the development and rupture of IA. Further research is needed to explore the underlying mechanisms and to validate these findings in larger and more diverse populations.

## Conclusions

To our knowledge, this study represents the first MR analysis investigating the causal relationship between hematologic traits and the risk of aSAH and uIA in the European population. Our findings suggests that the monocyte percentage of white cells may be a causative factor for the risk of aSAH. This discovery enhances our understanding of the pathogenesis of IA and the associated risk of aSAH, while also offering a potential hematologic indicator for monitoring individuals with uIA to mitigate their risk of aSAH.

### Supplementary Information

Supplementary Tables.

## Data Availability

Summary-level statistics of 25 hematologic traits were obtained from a GWAS study published by William et al. Summary-level statistics of aSAH and uIA were collected from a GWAS meta-analysis study published by Bakker et al., and the FinnGen (https://www.finngen.fi/en). All data in this study can be provided by the corresponding author Dr. Xuejun Li.
