# Supplementary material for: The association between hematologic traits and aneurysm-related subarachnoid hemorrhage: a two-sample mendelian randomization study
Source: Sci Rep. 2024 May 22;14:11694. doi: 10.1038/s41598-024-62761-1 (PMC11111675; doi:10.1038/s41598-024-62761-1)
Supplement: Supplementary file 1 — Supplementary Tables. [file 41598_2024_62761_MOESM1_ESM.docx]

Table S1. Other methods of MR analyses of Hematocrit, Hemoglobin concentration, RBC count, Basophil counts, eosinophil percentage of white cells, and Monocyte percentage of white cells on the risk of aSAH in the preliminary MR analyses.

| Exposure | Method | P-value | OR | LCI95 | UCI95 |
| --- | --- | --- | --- | --- | --- |
| Hematocrit | MR Egger | 6.79E-01 | 1.09 | 0.72 | 1.65 |
|  | Weighted median | 6.75E-01 | 0.93 | 0.68 | 1.28 |
|  | Simple mode | 5.32E-02 | 0.51 | 0.26 | 1.00 |
|  | Weighted mode | 8.10E-01 | 1.04 | 0.74 | 1.46 |
| Hemoglobin concentration | MR Egger | 5.64E-01 | 0.89 | 0.60 | 1.32 |
|  | Weighted median | 4.44E-01 | 0.89 | 0.67 | 1.19 |
|  | Simple mode | 8.29E-01 | 0.93 | 0.48 | 1.81 |
|  | Weighted mode | 6.26E-01 | 0.91 | 0.64 | 1.31 |
| RBC# | MR Egger | 6.60E-01 | 1.06 | 0.82 | 1.38 |
|  | Weighted median | 8.16E-01 | 0.97 | 0.78 | 1.22 |
|  | Simple mode | 4.13E-01 | 0.84 | 0.56 | 1.27 |
|  | Weighted mode | 8.92E-01 | 0.98 | 0.79 | 1.23 |
| Basophil# | MR Egger | 3.68E-01 | 0.74 | 0.38 | 1.42 |
|  | Weighted median | 6.29E-02 | 0.67 | 0.44 | 1.02 |
|  | Simple mode | 3.06E-01 | 0.68 | 0.33 | 1.40 |
|  | Weighted mode | 3.06E-01 | 0.77 | 0.47 | 1.25 |
| Eosinophil% | MR Egger | 2.46E-01 | 1.28 | 0.85 | 1.93 |
|  | Weighted median | 2.29E-01 | 1.17 | 0.90 | 1.52 |
|  | Simple mode | 4.91E-01 | 1.21 | 0.71 | 2.05 |
|  | Weighted mode | 4.74E-01 | 1.16 | 0.77 | 1.76 |
| Monocyte% | MR Egger | 6.09E-01 | 1.08 | 0.81 | 1.43 |
|  | Weighted median | 8.10E-01 | 1.03 | 0.83 | 1.27 |
|  | Simple mode | 4.83E-01 | 1.19 | 0.74 | 1.91 |
|  | Weighted mode | 9.79E-01 | 1.00 | 0.77 | 1.29 |

**#:** counts, **%**: percentage of white cells.

| Table S2. The sensitivity analyses of 25 hematologic traits on aSAH in the preliminary MR analyses | | | | | |
| --- | --- | --- | --- | --- | --- |
|  | exposure | Cochran's *Q* | | MR-Egger regression | |
|  |  | Q value | *P*-heterogeneity | Egger-intercept | *p*-egger |
| red blood cell | Hematocrit | 64.73571 | 2.83E-01 | -0.01474071 | 6.41E-02 |
|  | Hemoglobin concentration | 75.40509 | 2.51E-01 | -0.00695596 | 3.55E-01 |
|  | HLSR# | 113.3921 | 1.53E-01 | 0.01056916 | 8.63E-02 |
|  | HLSR/RBC | 124.9372 | 6.12E-02 | 0.01144048 | 6.32E-02 |
|  | IFR | 85.33579 | 6.48E-02 | 0.007712128 | 2.99E-01 |
|  | MCH | 134.6374 | 1.14E-01 | 0.001251117 | 8.07E-01 |
|  | MCH concentration | 26.76507 | 7.70E-01 | 0.01705699 | 1.10E-01 |
|  | MCV | 139.0578 | 8.03E-02 | 0.005465125 | 2.98E-01 |
|  | RBC# | 93.99455 | 4.23E-01 | -0.01004433 | 6.50E-02 |
|  | Reticulocyte# | 105.5663 | 7.47E-02 | -0.002420653 | 7.23E-01 |
|  | RF/RBC | 112.7018 | 1.04E-01 | 0.005873923 | 3.67E-01 |
| white blood cell | WBC# | 92.90991 | 4.83E-01 | 0.0124404 | 7.06E-02 |
|  | Basophil# | 25.14854 | 8.65E-01 | -0.001247129 | 9.10E-01 |
|  | Eosinophil# | 93.81754 | 4.28E-01 | 0.007752973 | 2.77E-01 |
|  | Lymphocyte# | 112.046 | 9.88E-02 | -0.00042237 | 9.63E-01 |
|  | Monocyte# | 102.7663 | 3.51E-01 | 7.10093E-07 | 1.00E+00 |
|  | Neutrophil# | 94.76537 | 8.27E-02 | 0.004348251 | 5.69E-01 |
|  | Basophil% | 22.53306 | 7.10E-01 | -0.01165831 | 3.18E-01 |
|  | Eosinophil% | 103.1062 | 0.0575 | -0.00185219 | 0.828 |
|  | Neutrophil% | 91.92539 | 6.65E-02 | -0.008036117 | 4.12E-01 |
|  | Monocyte% | 107.9602 | 1.38E-01 | 0.005844024 | 3.71E-01 |
| platelet | Platelet# | 136.6296 | 5.68E-02 | 0.004923849 | 3.96E-01 |
|  | PDW | 53.89316 | 8.55E-02 | -0.009315626 | 4.12E-01 |
|  | Plateletcrit | 143.722 | 1.09E-01 | 0.0127471 | 2.12E-02 |
|  | MPV | 134.1361 | 6.68E-02 | -0.007584398 | 1.40E-01 |

RBC: red blood cell traits, HLSR: high light scatter reticulocyte, HLSR/RBC: high light scatter reticulocyte percentage of red cells, IFR: immature fraction of reticulocytes, MCH: mean corpuscular hemoglobin, MCV: mean corpuscular volume, RF/RBC: reticulocyte fraction of red cells, PDW: platelet distribution width, MPV: mean platelet volume**, #:** counts, **%**: percentage of white cells.

| Table S3. The sensitivity analyses of 25 hematologic traits on uIA in the preliminary MR analyses | | | | | |
| --- | --- | --- | --- | --- | --- |
|  | exposure | Cochran's *Q* | | MR-Egger regression | |
|  |  | Q value | *P*-heterogeneity | Egger-intercept | *p*-egger |
| red blood cell | Hematocrit | 65.93325 | 3.43E-01 | -0.008570272 | 4.82E-01 |
|  | Hemoglobin concentration | 78.09197 | 1.67E-01 | 0.01423902 | 2.35E-01 |
|  | HLSR# | 108.5445 | 2.19E-01 | 0.006048509 | 5.17E-01 |
|  | HLSR/RBC | 125.0421 | 6.04E-02 | 0.005326491 | 5.73E-01 |
|  | IFR | 77.51253 | 3.07E-01 | 0.0135304 | 1.83E-01 |
|  | MCH | 122.9016 | 2.90E-01 | 0.006734301 | 3.71E-01 |
|  | MCH concentration | 32.52247 | 3.92E-01 | 0.009958478 | 5.57E-01 |
|  | MCV | 139.0594 | 9.02E-02 | 0.01507081 | 5.87E-02 |
|  | RBC# | 99.50922 | 2.78E-01 | -0.01108298 | 2.09E-01 |
|  | Reticulocyte# | 93.58651 | 2.70E-01 | 0.002914404 | 7.68E-01 |
|  | RF/RBC | 107.061 | 1.87E-01 | -0.001877684 | 8.48E-01 |
| white blood cell | WBC# | 105.8151 | 2.11E-01 | 0.000962932 | 9.31E-01 |
|  | Basophil# | 35.55481 | 3.95E-01 | -0.000910759 | 9.58E-01 |
|  | Eosinophil# | 93.97058 | 4.81E-01 | -0.003057516 | 7.72E-01 |
|  | Lymphocyte# | 93.97326 | 5.11E-01 | -0.000150415 | 9.90E-01 |
|  | Monocyte# | 93.99011 | 5.96E-01 | -0.01705965 | 6.00E-02 |
|  | Neutrophil# | 87.84898 | 1.66E-01 | -0.008249296 | 4.75E-01 |
|  | Basophil% | 36.30965 | 1.09E-01 | 0.01419306 | 4.88E-01 |
|  | Eosinophil% | 86.37298 | 0.3208661 | -0.004824246 | 0.6858815 |
|  | Neutrophil% | 82.5239 | 2.58E-01 | -0.00739155 | 6.02E-01 |
|  | Monocyte% | 73.03033 | 9.38E-01 | -0.003917092 | 6.73E-01 |
| platelet | Platelet# | 122.965 | 2.46E-01 | 0.003080516 | 7.11E-01 |
|  | PDW | 44.35387 | 3.32E-01 | -0.01868162 | 2.34E-01 |
|  | Plateletcrit | 125.7773 | 4.39E-01 | 0.01310048 | 1.02E-01 |
|  | MPV | 122.3838 | 1.80E-01 | -0.01140508 | 1.34E-01 |

RBC: red blood cell traits, HLSR: high light scatter reticulocyte, HLSR/RBC: high light scatter reticulocyte percentage of red cells, IFR: immature fraction of reticulocytes, MCH: mean corpuscular hemoglobin, MCV: mean corpuscular volume, RF/RBC: reticulocyte fraction of red cells, PDW: platelet distribution width, MPV: mean platelet volume**, #:** counts, **%**: percentage of white cells.

Table S4. Other methods of MR analyses of Hematocrit, Hemoglobin concentration, Basophil counts, and Monocyte percentage of white cells on the risk of aSAH in the replicated MR analyses

| Exposure | Method | P-value | OR | LCI95 | UCI95 |
| --- | --- | --- | --- | --- | --- |
| Hematocrit | MR Egger | 6.38E-01 | 0.88 | 0.53 | 1.48 |
|  | Weighted median | 2.10E-01 | 1.23 | 0.89 | 1.71 |
|  | Simple mode | 6.69E-01 | 1.17 | 0.57 | 2.42 |
|  | Weighted mode | 2.95E-01 | 1.26 | 0.82 | 1.94 |
| Hemoglobin concentration | MR Egger | 7.79E-01 | 0.94 | 0.59 | 1.48 |
|  | Weighted median | 3.70E-01 | 1.15 | 0.84 | 1.58 |
|  | Simple mode | 5.98E-01 | 0.81 | 0.37 | 1.76 |
|  | Weighted mode | 9.27E-01 | 1.02 | 0.66 | 1.58 |
| Basophil# | MR Egger | 8.30E-01 | 0.59 | 0.59 | 1.95 |
|  | Weighted median | 9.23E-01 | 0.68 | 0.68 | 1.53 |
|  | Simple mode | 4.82E-01 | 0.26 | 0.26 | 1.89 |
|  | Weighted mode | 6.34E-01 | 0.83 | 0.39 | 1.77 |
| Monocyte% | MR Egger | 6.09E-01 | 1.08 | 0.81 | 1.43 |
|  | Weighted median | 8.08E-01 | 1.03 | 0.83 | 1.26 |
|  | Simple mode | 4.78E-01 | 1.19 | 0.74 | 1.90 |
|  | Weighted mode | 9.80E-01 | 1.00 | 0.76 | 1.30 |

**#:** counts, **%**: percentage of white cells.

Table S5. The sensitivity analyses of 4 selected hematologic traits on aSAH in the replicated MR analyses

|  | exposure | Cochran's *Q* | | MR-Egger regression | |
| --- | --- | --- | --- | --- | --- |
|  |  | Q value | *P*-heterogeneity | Egger-intercept | *p*-egger |
| red blood cell | Hematocrit | 116.2204 | 1.94E-01 | -0.009206782 | 2.72E-01 |
|  | Hemoglobin concentration | 121.0673 | 2.22E-01 | -0.005530314 | 4.60E-01 |
| white blood cell | Basophil# | 79.05925 | 5.02E-02 | -0.02055171 | 5.85E-02 |
|  | Monocyte% | 177.5421 | 2.39E-01 | 0.000553728 | 9.18E-01 |

**#**: counts, **%**: percentage of white cells.
